# Neuropathic Pain Secondary to Multiple Sclerosis: A Narrative Review

**DOI:** 10.7759/cureus.61587

**Published:** 2024-06-03

**Authors:** Billy McBenedict, Kang Suen Goh, Ryan Chun Chien Yau, Sara Elamin, Walaa H Yusuf, Gabriel Verly, Anusha Thomas, Berley Alphonse, Kaoutar Ouabicha, Gabriella Valentim, Wilhelmina N Hauwanga, Bruno Lima Pessôa

**Affiliations:** 1 Neurosurgery, Fluminense Federal University, Niterói, BRA; 2 Internal Medicine, Monash University Malaysia, Johor Bahru, MYS; 3 Medicine, University of Medical Sciences and Technology, Khartoum, SDN; 4 Medicine, Mansoura University, Mansoura, EGY; 5 Neurology, Federal University of Rio de Janeiro, Rio de Janeiro, BRA; 6 Neurology, Christian Medical College & Hospital, Ludhiana, IND; 7 Internal Medicine, University Notre Dame of Haiti, Port-au-Prince, HTI; 8 Medicine, Sidi Mohammed Ben Abdellah University, Fes, MAR; 9 Nursing, Fluminense Federal University, Niterói, BRA; 10 Family Medicine, Federal University of Rio de Janeiro, Rio de Janeiro, BRA

**Keywords:** multidisciplinary approaches, demyelination, central sensitization, neuropathic pain, multiple sclerosis

## Abstract

Multiple sclerosis (MS) is a chronic autoimmune disease that affects the central nervous system (CNS). Neuropathic pain in MS is a debilitating symptom that significantly impairs the quality of life for a substantial proportion of MS patients. Neuropathic pain in MS stems primarily from demyelination, axonal loss, CNS inflammation, and direct damage to the myelin sheath, leading to pain manifestations such as ongoing extremity pain, Lhermitte's phenomenon, and trigeminal neuralgia (TN). The pathophysiological mechanisms behind MS-related neuropathic pain are explored in this review, highlighting central sensitization, neural dysfunction, spinal thalamic tract dysfunction, and inflammatory processes that exacerbate neuronal damage. Neuropathic pain in MS necessitates comprehensive assessment tools and neurophysiological tests to differentiate neuropathic pain from other MS symptoms accurately. Treatment strategies for MS-related neuropathic pain encompass pharmacological interventions, including anticonvulsants and antidepressants, and emerging therapies targeting specific inflammatory processes. The review advocates for a holistic approach to management, incorporating innovative treatments and multidisciplinary strategies to address both the physical symptoms and psychosocial aspects of this disorder. This comprehensive overview underscores the importance of ongoing research into targeted therapies to improve patient outcomes and enhance the quality of life for those affected by MS.

## Introduction and background

Neuropathic pain in multiple sclerosis (MS) arises from nervous system damage and manifests diversely, including ongoing extremity pain and Lhermitte’s phenomenon. Its prevalence among MS patients ranges from 26% to 58% [1]. Diagnosis involves comprehensive assessment tools such as the Douleur Neuropathique en 4 Questions and neurophysiological tests such as somatosensory-evoked potentials, laser-evoked potentials, and, more recently, contact heat-evoked potentials [1,2]. This pain type encompasses various sensations, such as burning or electric-like sensations triggered by movement, often accompanied by allodynia, hyperalgesia, or hypoalgesia. Peripheral neuropathic pain may coexist with nociceptive or central pain, complicating classification. Additionally, MS-related headaches may involve neuropathic, inflammatory, and musculoskeletal components, warranting multidimensional treatment approaches. Female patients and those with longer disease duration and higher disability levels are more prone to neuropathic pain, and evaluation for neuropathic pain is crucial for all MS patients [3].

Neuropathic pain and nociceptive pain

Neuropathic pain is defined as pain initiated or caused by a primary lesion or dysfunction in the nervous system. This type of pain encompasses a range of disease states and presents with various symptoms. In contrast, nociceptive pain is defined as pain induced by an external noxious stimulus to a structurally and functionally intact nervous system. This distinction is crucial for differentiating between neuropathic and nociceptive pain [4]. Neuropathic pain is often described as having a lancinating or continuous burning quality and is frequently associated with abnormal sensory signs such as allodynia (pain resulting from a stimulus that does not normally provoke pain) or hyperalgesia (an increased response to a normally painful stimulus). It may also present spontaneously as dysesthesia, commonly observed with thalamic lesions. These sensory phenomena can be further characterized into static (chronic) or dynamic (episodic or paroxysmal) subtypes. Mechanistically, allodynia implies that elements of the sensory nervous system, which typically signal innocuous sensations, have started to encode painful stimuli, whereas hyperalgesia indicates that the structures involved in nociception have become hyperexcitable [4]. Spasticity may alter sensory perception, leading to abnormal sensations such as tingling, burning, or pins-and-needles, which can contribute to neuropathic pain and functional pain syndromes [5]. Prolonged spasticity can lead to central sensitization, a process where the central nervous system (CNS) becomes hypersensitive to pain signals. This can result in heightened pain perception and the development of chronic pain syndromes [6].

Longer disease duration and greater disability scores, such as higher Expanded Disability Status Scale scores, are associated with an increased risk of neuropathic pain in MS patients [7]. The presence of brain or spinal cord lesions, particularly in regions associated with sensory processing and pain modulation, may increase the likelihood of neuropathic pain in MS [8]. Younger age at MS onset has been identified as a risk factor for neuropathic pain development, possibly due to differences in disease course and neuroplasticity [9]. Comorbid depression and anxiety are common in MS patients and have been associated with an increased risk of neuropathic pain [7]. Certain disease-modifying therapies and symptomatic treatments used in MS management, such as interferon-beta and corticosteroids, may contribute to neuropathic pain development as adverse effects [7].

Traditionally, nociceptive pain is believed to arise from the application of a noxious stimulus to a structurally intact nervous system. In contrast to neuropathic pain, there is relatively scant literature available on the mechanisms of nociceptive pain. This is primarily because animal models designed to study pain mechanisms almost always involve neural tissue injury induced mechanically, chemically, or thermally. Sorkin and Wallace recently discussed the mechanisms of acute pain, which we interpret as referring to nociceptive pain [4]. The stages in the nociceptive pain mechanism are similar to those of peripheral neuropathic pain. They include peripheral sensitization at sensory nerve endings and the dorsal root ganglia, followed by central sensitization at the dorsal horn, influenced by descending brainstem pathways, culminating in the final perception of pain at the sensory cortex. A fundamental difference between nociceptive pain and neuropathic pain is their response to opioids; nociceptive pain responds well to opioids, whereas neuropathic pain does not. This difference may be due to the loss of peripheral opioid effects, loss of spinal opioid receptors, and increased activity of physiological opioid antagonists. Interestingly, spinal opioid analgesics offer greater pain relief than systemic administration in neuropathic pain, and cannabinoid analgesia appears more effective than opioid analgesia [4].

Nociceptive pain arises from the activation of nociceptors in response to tissue damage or inflammation. Peripheral sensitization, involving the release of inflammatory mediators, and central sensitization, characterized by increased excitability of dorsal horn neurons, play key roles. Examples of nociceptive pain syndromes are osteoarthritis, rheumatoid arthritis, and postoperative pain [10]. Neuropathic pain results from damage or dysfunction of the nervous system and is characterized by abnormal sensory processing. Examples include diabetic neuropathy, postherpetic neuralgia, and phantom limb pain [11,12]. Visceral pain arises from the activation of nociceptors in internal organs.

The mechanisms involve visceral afferent sensitization, viscerosomatic convergence, and central sensitization in the spinal cord and brain. Visceral pain syndrome involves irritable bowel syndrome, pelvic pain disorders, and pancreatitis [13]. Musculoskeletal pain involves nociceptive input from muscles, bones, ligaments, and tendons. The mechanisms include tissue injury, inflammation, muscle spasm, and sensitization of peripheral and central pain pathways. Musculoskeletal pain syndrome includes fibromyalgia, myofascial pain syndrome, and low back pain [14,15]. Central sensitization syndromes involve the amplification of pain signaling within the CNS.

The mechanisms include increased synaptic transmission, reduced inhibitory control, and neuroplastic changes in the spinal cord and brain. Central sensitization syndromes involve fibromyalgia, chronic fatigue syndrome, and temporomandibular joint disorder [10,16]. Mixed pain syndromes may involve a combination of nociceptive, neuropathic, visceral, and/or central sensitization components. Mechanisms vary depending on the specific combination of pain types present. Examples include complex regional pain syndrome, chronic pelvic pain syndrome, and post-stroke pain [10,11].

Despite advancements in understanding MS, the management of neuropathic pain remains challenging due to its complex and multifactorial nature. Treatments often provide limited relief and are associated with significant side effects, necessitating a comprehensive and updated synthesis of recent research. This review aims to bridge the gap by consolidating the latest findings on the pathophysiology, clinical manifestations, diagnostic approaches, and management strategies for neuropathic pain in MS. By providing a thorough analysis of the current landscape, this review seeks to enhance understanding and management of neuropathic pain in MS patients, ultimately improving their quality of life.

## Review

Pathophysiology of neuropathic pain in MS

Although pain is a common symptom across various conditions, the underlying pathophysiologies differ significantly. Truini et al. suggest that the mechanisms responsible for pain syndromes associated with multiple MS remain largely theoretical [15]. For example, ongoing extremity pain is hypothesized to result from thalamic or cortical deafferentation due to multiple lesions along the spinothalamocortical pathways and is often described as a burning sensation [15,16]. In contrast, trigeminal neuralgia (TN) is believed to arise from high-frequency discharges caused by a combination of intra-axial inflammatory demyelination and extra-axial mechanical demyelination of the trigeminal primary afferents [9]. This damage disrupts the spinothalamic-thalamocortical pathways, contributing to central sensitization and heightened pain system excitability. Dysfunction of these pathways, particularly the spinothalamocortical tract, accompanies the development of central pain in MS, although other factors, such as lesions in the thalamus and inflammatory lesions, also play a role. TN secondary to MS resembles classical and idiopathic TN, featuring sudden, unilateral, stabbing, or electrical shock-like pain in the distribution of the fifth cranial nerve. Stimulus dependence is a hallmark, but spontaneous attacks occur with variable frequency and unclear remission patterns [17]. The role of stress as an MS trigger remains debated, with empirical studies showing mixed results. While reducing distress may decrease proinflammatory cytokine production in MS patients, no direct biological mechanism linking stress and inflammation has been confirmed.

Different mechanisms are proposed for various MS-related pain syndromes. Lhermitte’s phenomenon is attributed to high-frequency discharges originating from demyelination of the dorsal column primary afferents, typically triggered by neck flexion [9,18]. Painful tonic spasms (PTS) are thought to result from high-frequency discharges in the corticospinal pathways, leading to muscle contractions and ischemic pain. These spasms may be triggered by touch, movement, hyperventilation, or emotions and are often preceded by a "somesthetic aura" [9,19]. Spasticity pain, a prevalent symptom, is theorized to arise from disinhibition due to a corticospinal tract lesion, which enhances the tonic stretch reflex, leading to excessive muscular activity and mechanical muscle pain [9].

It is crucial to note that various pain modalities in MS are linked to the specific locations of demyelinating plaques. For instance, TN is likely associated with plaques in the pons, Lhermitte’s phenomenon with plaques in the cervical dorsal columns, and migraines with plaques in the midbrain or periaqueductal gray matter [8,9]. Recognizing these associations is essential for improving the diagnosis and treatment of pain in MS patients. Plaques located near the brain's ventricles, particularly in the periventricular white matter regions, are common in MS. These plaques can disrupt the transmission of neural signals along pain pathways, leading to various types of pain experienced by individuals with MS [20]. Plaques affecting the spinal cord are frequently observed in MS. These plaques can interfere with the transmission of sensory signals between the body and the brain, potentially contributing to neuropathic pain. This can manifest as sensations of tingling, burning, or electric shock-like pain [18]. In addition to white matter lesions, cortical gray matter involvement is increasingly recognized in MS. Cortical lesions, particularly in regions associated with pain processing, such as the somatosensory cortex and insula, may contribute to the development of central neuropathic pain in MS [21].

The specific plaque locations in the CNS are implicated in lesions inducing abnormal impulses through motor axons or causing acquired sodium channelopathy in injured nerves. Demyelinated axons can exhibit changes in the distribution and expression of ion channels (e.g., sodium and calcium channels). These alterations can increase neuronal excitability and contribute to the generation and maintenance of pain signals. The role of sodium channels in neuropathic pain associated with MS is significant and multifaceted. Neuropathic pain is a common and debilitating symptom experienced by many individuals with MS, and sodium channels play a crucial role in its pathophysiology [22]. The attributed mechanisms include increased sodium channel expression and function and conduction abnormalities. In MS, demyelination exposes axons and leads to an upregulation of voltage-gated sodium channels (VGSCs) along demyelinated axons. This dysregulation results in increased sodium channel expression and function, particularly Nav1.7, Nav1.8, and Nav1.9 subtypes. The heightened sodium channel activity contributes to neuronal hyperexcitability and aberrant firing of action potentials, leading to the generation and propagation of neuropathic pain signals [22]. Demyelination disrupts saltatory conduction, which normally occurs in myelinated axons, leading to inefficient signal propagation along nerve fibers. Sodium channels play a pivotal role in action potential generation and propagation, and their dysregulation exacerbates conduction abnormalities in demyelinated axons, contributing to neuropathic pain in MS [22].

Given the central role of sodium channels in neuropathic pain associated with MS, they represent promising therapeutic targets for pain management. Sodium channel blockers, such as carbamazepine, phenytoin, and lidocaine, have demonstrated efficacy in alleviating neuropathic pain symptoms by dampening neuronal hyperexcitability and reducing aberrant firing of action potentials [23]. In neuropathic pain, Nav1.7, in particular, has garnered attention as a key player in neuropathic pain mechanisms. Nav1.7 is a subtype of VGSCs encoded by the *SCN9A* gene. It plays a critical role in the initiation and propagation of action potentials in neurons. Nav1.7 channels are upregulated in sensory neurons in MS patients experiencing neuropathic pain [21]. Selective blockade or modulation of Nav1.7 channels holds promise as a targeted therapeutic strategy for managing neuropathic pain in MS, although further research is needed to elucidate its specific role and therapeutic potential [21]. Autoimmune activity in MS results in myelin damage and microglial hyperactivity, triggering a reactive immune response contributing to central pain development. Proinflammatory cytokines released due to immune activity disrupt the blood-brain barrier and affect nervous system function, potentially leading to hyperalgesia. Dysregulated glutamate homeostasis in MS further contributes to neuropathic pain by promoting demyelination and axonal damage [23].

Studies highlight the intricate interplay between neurons and supportive cells, such as astrocytes and microglia, as well as the dynamic interactions between neurons and immune cells, such as T-cells and macrophages, in the development of chronic neuropathic pain (CNP) [24]. This suggests that toll-like receptors found in immune and glial cells, in addition to sensory neurons, not only detect nociceptive signals from tissue injury but also modulate the complex network of interactions between neurons and other cellular elements [25]. Glial cell involvement in the immune response to CNS inflammation may also contribute to pain development [8]. Lesions in the CNS, especially in the spinal cord and brainstem, are associated with central neuropathic pain syndromes, with TN likely originating from demyelination at the trigeminal nerve root entry zone in the pons [8,26].

Microglia, implicated in MS pathology, may play a significant role in central pain initiation, particularly in the spinal cord, making them promising targets for MS-related neuropathic pain therapy. Microglial cells are specialized macrophages within the CNS that play a pivotal role in the innate immunity of the spinal cord and brain. These cells are crucial not only for their protective functions against invaders, microbes, demyelination, and trauma but also for removing defective cells and neurons. In neuropathic pain conditions, microglia contribute to the recruitment and activation of other immune cells, thereby exacerbating the inflammatory response. They possess a wide array of receptors and chemical mediators that enable rapid and precise communication with every nerve tissue cell, ensuring robust immune system protection.

**Figure 1:**
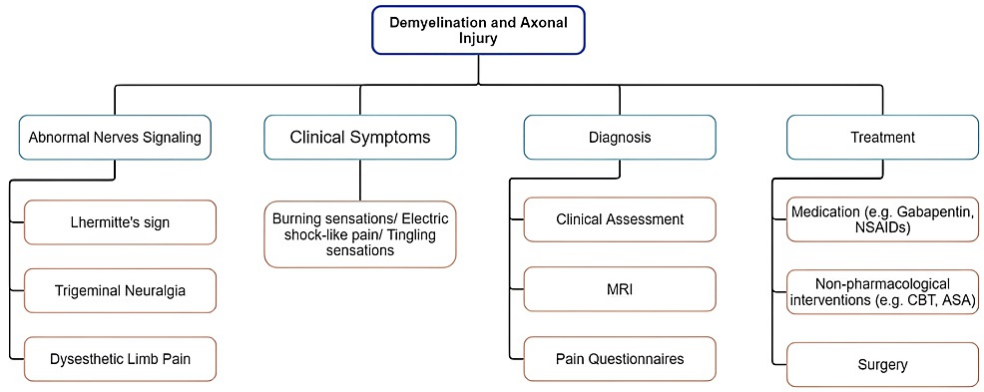
Mind map exploring the etiology and management strategies. The mind map was constructed from the articles included in the review and generated using the Miro Mind Mapping tool [27-49].

Modern molecular genetics tools have elucidated the critical role of microglia in the development, maintenance, mediation, and progression of neuropathic pain. In neuropathic pain models, where peripheral or spinal nerves are compressed, pain is alleviated when microglial cell activity is inhibited [27]. Advanced research techniques, including transgenic and chimeric animal models of gene targeting, have shown that nerve damage activates microglia, leading to rapid changes in their morphology, migration rate, and proliferation [27]. A recent study used a novel chemogenetic approach to investigate microglial function in mice with neuropathic pain. By manipulating microglial Gi signaling through chemogenetic methods, researchers were able to reduce chronic pain by inhibiting neuroinflammation in transgenic mice (Figure 1) [27].

Clinical features and classification of neuropathic pain in MS ​​​patients

MS is characterized by diverse neurological symptoms, including sensory loss, visual disturbances, muscle weakness, impaired balance, dysesthetic pain, and TN, with dysesthetic extremity pain particularly prevalent in the legs and feet [28]. Initially following a relapsing-remitting course, MS transitions to a progressive phase later on. Pain in MS can be stimulus-independent (persistent or paroxysmal) or evoked (hyperalgesia and allodynia) [28,29]. Other MS-related pain manifestations include Lhermitte’s phenomenon, PTS, spasticity pain, optic neuritis pain, musculoskeletal pain from postural anomalies, and headaches, especially migraines [28,29]. Dysesthetic pain in MS can be influenced by temperature changes, touch, activity, and stress, often worsening when fatigued or at night [28,29].

The proposed classification of pain in patients with MS is primarily based on phenomenology and includes hypotheses about the underlying pathophysiologic mechanisms. These hypotheses may ultimately serve as the foundation for a mechanism-based classification of pain in MS [19].

Continuous Central Neuropathic Pain

MS causes demyelination and axonal damage in the brain and spinal cord, leading to mechanisms hypothesized to cause central neuropathic pain. MS lesions that induce central hyperexcitability and disrupt spinothalamic pathways may account for continuous central extremity pain. This type of damage in MS appears to result in disinhibition, particularly affecting the “cold inhibition of pain,” leading to continuous pain described as burning and/or throbbing. Patients with MS generally describe this as dysesthetic extremity pain. Since central pain in MS often manifests bilaterally in the lower extremities, the causative lesion is likely located in the spinal cord in many cases [19].

Intermittent Central Neuropathic Pain

Demyelination in patients with MS can cause ectopic impulses in damaged neurons, leading to ephaptic transmission to normally conducting neurons. Depending on the location of the lesions, such abnormalities in the somatosensory pathways may produce intermittently painful conditions in MS patients, including TN and Lhermitte’s sign [19].

Musculoskeletal Pain

Lesions affecting motor neurons can result in the involuntary, intermittent, painful muscle contractions characteristic of paroxysmal tonic spasms (PTS). Although this type of pain is caused by demyelination, it would not be classified as neuropathic pain under current diagnostic criteria because the nerve damage does not appear to affect the somatosensory pathways. By causing weakness, muscle spasms, spasticity, and reduced mobility, MS predisposes patients to develop secondary musculoskeletal pain, particularly in the lower back. Additionally, treatments for MS can contribute to secondary musculoskeletal pain. For example, interferon-beta often causes myalgias, and chronic steroid use can lead to osteoporosis, potentially resulting in vertebral compression fractures [19].

Mixed Neuropathic and Non-neuropathic Pain

Some types of chronic pain associated with MS do not readily fit into the previously mentioned categories, with headaches (including migraines) being a prevalent example. In patients with MS, headaches likely involve a complex mixture of neuropathic, inflammatory, and musculoskeletal mechanisms [19].

Diagnostic criteria for chronic central neuropathic pain in MS

The diagnostic criteria for chronic central neuropathic pain in MS presented in this section are adapted from Finnerup et al. (2016) [12]. The AAPT (ACTTION-American Pain Society Pain Taxonomy) core diagnostic criteria for chronic central neuropathic pain in MS encompass multiple dimensions to provide a comprehensive framework for diagnosis and management. Each dimension addresses a different aspect of the pain experience, ensuring a holistic understanding and approach to treatment.

Dimension 1: Core Diagnostic Criteria

This dimension includes the essential diagnostic criteria required to identify chronic central neuropathic pain in MS. It outlines the fundamental characteristics and clinical features necessary for a definitive diagnosis, ensuring that the diagnosis is accurate and standardized as described [12]: (1) diagnostic evaluation confirming MS in which the patient fulfills McDonald criteria for MS (DIS = disseminated in space, DIT = disseminated in time, or PPMS = primary progressive MS) [12]; (2) continuous or recurrent pain after MS, with onset after established diagnosis of MS; (3) pain duration of at least three months; (4) pain is described within the area of the body affected by an MS lesion in the brain or spinal cord; (5) pain is associated with sensory changes in the same neuroanatomically plausible distribution, as indicated by the presence of at least one positive sensory sign (e.g., dynamic mechanical or cold allodynia) or one negative sensory sign (e.g., elevated thresholds to cold or warm or decreased sensation to touch, pinprick, or thermal stimuli); and (6) there is no other diagnosis that better explains the pain.

Dimension 2: Common Features

This dimension describes the typical clinical presentations and symptoms commonly associated with chronic central neuropathic pain in MS. It helps clinicians recognize the common patterns of pain and related symptoms, facilitating better identification and management of the condition. Central pain in MS shares features with other central pain conditions. It can be constant or intermittent and sometimes evoked by stimuli (e.g., touch-evoked allodynia). MS-related central neuropathic pain is often described with terms such as "burning," "pricking," "squeezing," "tingling," and "aching." It is typically associated with abnormal sensory signs such as numbness and paresthesia. TN, a paroxysmal facial pain in MS patients, has an abrupt onset lasting a few seconds and is usually triggered by innocuous stimuli or movement, though it can also occur spontaneously. Some patients experience pain between episodes. In MS, TN can be unilateral or bilateral, known as secondary TN. Lhermitte's phenomenon is a brief, transient electric shock-like sensation often felt in the back and triggered by neck movement, commonly associated with MS [12,30].

Dimension 3: Common Medical and Psychiatric Comorbidities

This dimension addresses the medical and psychiatric conditions frequently found in conjunction with chronic central neuropathic pain in MS. Understanding these comorbidities is crucial for comprehensive patient care, as they can significantly impact the overall health and treatment outcomes of patients. The AAPT highlights the significant impact of psychosocial factors on the overall experience of chronic pain, considering them as potential risk factors, protective factors, or comorbidities. Conditions such as spasticity, paresis, musculoskeletal pain, and dizziness can exacerbate pain and complicate the diagnosis and treatment of central pain in MS. Additionally, fatigue and cognitive impairment, common in MS, significantly reduce patients’ quality of life. Mental health comorbidities such as depression, anxiety, and bipolar disorder further contribute to disability in MS. Therefore, various medical issues beyond pain can significantly influence pain severity and quality of life in people with MS and should be considered in the overall evaluation of pain in this population. An unexplored area is the potential impact of disease-modifying drugs on pain in MS patients. These immune-modulating and immune-suppressing agents may, due to their adverse effects, also affect the general well-being of patients and indirectly influence pain [12,30].

Dimension 4: Neurobiological, Psychosocial, and Functional Consequences

This dimension examines the broader impact of chronic central neuropathic pain on the patient's neurobiology, psychosocial status, and daily functioning. It highlights how pain affects brain function, mental health, social interactions, and the ability to perform everyday activities, emphasizing the need for a multidisciplinary approach to treatment. Central pain in MS, like other chronic pain conditions, is associated with significant psychosocial and functional consequences. In MS, there is a complex interaction between pain, depression, sleep dysfunction, and fatigue. Pain severity and depression are key factors influencing pain interference in this population, even though these findings are not limited to central pain alone [12,30].

Dimension 5: Putative Neurobiological and Psychosocial Mechanisms, Risk Factors, and Protective Factors

This dimension explores the underlying neurobiological and psychosocial mechanisms thought to contribute to chronic central neuropathic pain in MS. It also identifies potential risk factors that may predispose individuals to this type of pain and protective factors that could mitigate its development or severity. Understanding these mechanisms and factors can guide targeted interventions and preventative strategies. The mechanisms underlying central pain in MS are complex, involving central sensitization and changes related to deafferentation. The spinothalamocortical pathways are primarily affected, except for the Lhermitte phenomenon, which is related to dorsal column lesions. The primary pain mechanism in ongoing MS pain, whether ectopic firing in preserved pathways or deafferented neurons, remains unclear.

TN in MS is thought to result from high-frequency discharges due to demyelination of trigeminal primary afferents. Similarly, the Lhermitte phenomenon is believed to stem from ectopic discharges in demyelinated dorsal column afferents and is more common in younger patients. Nonspecific central pain is more prevalent in those with a progressive or progressive-relapsing course of MS. Patients with pain tend to be more disabled than those without. Psychosocial and demographic factors contribute to pain in MS, with psychological factors accounting for 25% of the variance in pain intensity in one study. However, specific risk factors for central pain in MS are not well understood. Further research is needed to determine the key mechanisms of central pain in MS conclusively [12,30]. The relevance of these dimensions in the AAPT core diagnostic criteria for chronic central neuropathic pain in MS lies in their comprehensive approach to diagnosis and management. By addressing each dimension, clinicians can (1) ensure accurate diagnosis by using standardized core diagnostic criteria to accurately identify chronic central neuropathic pain in MS, (2) recognize common features by understanding the typical clinical presentations to better identify and treat patients, (3) manage comorbidities by considering the impact of medical and psychiatric comorbidities for holistic patient care, (4) assess broader impact by evaluating the broader neurobiological, psychosocial, and functional consequences to tailor interventions, and (5) guide treatment by using insights into underlying mechanisms, risk factors, and protective factors to develop targeted and effective treatment plans.

Diagnosis of neuropathic pain in MS

The diagnostic criteria for neuropathic pain in MS emphasize lesion history, pain distribution, sensory changes, and imaging confirmation. Neurophysiological techniques and magnetic resonance imaging (MRI), particularly for assessing trigeminal reflexes and brain lesions, play crucial roles in diagnosing MS-related TN. While MRI is essential for MS diagnosis and detecting trigeminal nerve compression, diagnostic confirmation of neuropathic pain syndromes primarily relies on clinical symptoms. Painful dysesthesias, Lhermitte's sign, and PTS are common forms of central neuropathic pain in MS, often affecting various body regions. Musculoskeletal pain and headaches are also prevalent, while psychogenic pain can stem from impulses transmitted from the limbic system. Differentiating MS-related pain from other causes is crucial.

Various tools are employed for pain assessment, such as the Visual Analogue Scale, Numerical Rating Scale, Verbal Category Scale, Faces Pain Scale, Brief Pain Inventory, Douleur Neuropathique en 4 Questions, Neuropathic Pain Symptom Inventory, Leeds Assessment of Neuropathic Symptoms and Signs, and Beck’s Depression Inventory, alongside questionnaires such as McGill pain questionnaire and indirect scales for spasticity assessment [31-33]. These tools provide comprehensive analyses of pain severity, characteristics, and neuropathic pain symptoms. Additionally, assessing central neuropathic pain involves various tests, such as evaluating sensation thresholds and scrutinizing pain adaptation and offset analgesia [31-33]. While the tools offer valuable insights, additional assessments such as quantitative sensory testing or imaging are essential for a comprehensive evaluation.

MRI is pivotal for understanding neuropathic mechanisms in MS-related pain by assessing demyelinating lesion locations, particularly in regions such as the thalamus, and their correlation with pain symptoms.

Advanced techniques such as diffusion tensor imaging and MRI reveal cortical involvement in MS-related pain, shedding light on structural and functional changes in pain processing areas [26]. Functional methods complement structural imaging, pinpointing cerebral activity linked to central sensitization in chronic pain conditions, offering deeper insights into MS pathology and pain perception [26]. Despite limitations, ongoing neuroimaging advancements, especially MRI, hold promise for understanding the complex interplay between MS pathology and pain perception. Electrophysiological techniques and nerve biopsy samples contribute to assessing neuronal function attenuation and neuropathy extent. Various investigative tools, including advanced neuroimaging techniques, skin biopsy, microneurography, autonomic nervous system function assessment, reflexes assessment, and laser-evoked potentials, contribute to a comprehensive understanding of neurologic function in MS-related pain [26,34].

Treatment and management strategies

Recent research has categorized MS treatments by efficacy levels, with highly effective options including ocrelizumab, ofatumumab, natalizumab, alemtuzumab, and mitoxantrone [35]. However, these are disease-modifying treatments, not cures. Treatment strategies involve immunosuppressive agents, but caution is advised, especially for pregnant women [36]. Cannabinoids show promise for MS symptoms, particularly spasticity, but evidence for treating CNP remains inconclusive [37]. These medications target the endocannabinoid system and offer an alternative approach to pain management in MS. Conventional pain medications offer limited relief, with no single treatment providing significant pain reduction [38]. Antidepressants, particularly tricyclic antidepressants and serotonin-norepinephrine reuptake inhibitors such as duloxetine, play a crucial role in neuropathic pain management. However, their use is limited by side effects and uncertainty regarding efficacy in MS-associated CNP [38]. Anticonvulsants such as gabapentin and carbamazepine are commonly prescribed but may have limited effectiveness and tolerability [38].

Managing neuropathic pain necessitates a comprehensive approach encompassing accurate diagnosis and interdisciplinary collaboration, alongside setting realistic treatment objectives. Tailored treatments for various types of neuropathic pain in MS have been delineated in research [31]. Painful dysesthesias are typically addressed with tricyclic antidepressants, gabapentinoids, and newer antidepressants, with cannabinoids considered as a secondary option. Lhermitte's sign may be alleviated through intravenous lidocaine and mexiletine administration. PTS often respond to antiepileptic drugs, lidocaine infusions, and botulinum toxin injections. Musculoskeletal pain management typically begins with nonsteroidal anti-inflammatory drugs, followed by physiotherapy and physical modalities such as electrotherapy and cryotherapy. TN is commonly treated with carbamazepine as the first-line therapy, with oxcarbazepine, lamotrigine, and gabapentin as alternative options. Headaches associated with MS often benefit from standard migraine and tension headache treatments. Moreover, corticosteroids serve as the primary pharmacotherapy for pain in retrobulbar optic neuritis [31].

Non-pharmacological interventions play a significant role in managing MS symptoms. These interventions include neurostimulation therapy, exercise, psychological approaches, and complementary therapies such as acupuncture [39]. Evidence supporting transcutaneous electrical nerve stimulation, psychotherapy, transcranial stimulation, hydrotherapy, and reflexology is currently under construction [40]. Acupuncture, particularly Ashi scalp acupuncture, shows promise as a complementary therapy for TN in MS patients, offering pain relief without adverse effects [39]. In addition, surgical options such as microvascular decompression and trigeminal rhizotomy are preferred for patients with neurovascular contact, providing significant pain relief for the majority of patients even several years after treatment [40].

Radiofrequency thermocoagulation (RFT) offers pain relief by inducing mild hypoalgesia in affected trigeminal divisions without causing notable sensory deficits [41]. Type I TN typically responds better to RFT, whereas bilateral pain and psychiatric comorbidities correlate with poorer outcomes [41]. RFT proves effective for type II TN associated with MS but necessitates anesthesia induction in the affected trigeminal divisions. Its advantage lies in its precise targeting of trigeminal divisions compared to other percutaneous procedures. However, limited research has explored outcome predictors post-RFT [41]. Gamma Knife radiosurgery (GKRS) serves as a minimally invasive approach for treating refractory facial pain, predominantly TN [42]. GKRS boasts a low risk of facial paresthesias, approximately 80% significant pain relief rate, and low recurrence in initially relieved patients. Since its initial application by Lars Leksell in 1951, GKRS has become a primary treatment option for medically refractory TN [42,43].

Neuropathic pain poses significant challenges to quality of life across various dimensions. Physically, it can lead to discomfort, sharp pains, and numbness, limiting mobility and daily activities. Emotionally, it often triggers frustration, anxiety, and depression due to its persistent nature and unpredictable flare-ups, fostering emotional distress. Socially, it may isolate individuals, hindering participation in social activities and straining relationships. Sleep disturbances further compound the issue, with neuropathic pain disrupting sleep patterns and exacerbating pain levels, impacting overall well-being. Financially, managing neuropathic pain can be costly, with ongoing medical treatments and potential loss of income from limited work capacity. Moreover, cognitive functions may suffer, with pain-related distractions impairing attention, memory, and decision-making. Ultimately, individuals with neuropathic pain often report lower quality of life scores across both physical and mental health domains, highlighting the profound impact of this condition on overall well-being [39].

Pain in MS is associated with higher levels of disability, reduced participation in recreational activities, mood disturbances, and decreased enjoyment of life. MS patients report significantly higher levels of pain interference in daily life compared to reference subjects. Psychosocial factors contribute substantially to pain intensity and functioning in MS patients, highlighting the importance of addressing these factors in pain management. Pain severity in MS correlates strongly with reduced social functioning and mental health, particularly in women, and is linked to anxiety and depression. Additionally, QoL serves as a gauge of treatment effectiveness and can predict disease progression [44].

New therapeutic avenues are emerging for managing MS-related pain, complementing traditional treatments such as antidepressants and anticonvulsants. Recent research emphasizes targeting inflammation, with drugs focusing on the peripheral immune system and the mammalian target of rapamycin (mTOR).

Rapamycin, an mTOR inhibitor, has shown promise in reversing motor disease signs and alleviating pain in MS animal models by suppressing immune responses and inhibiting glial cell activation [28]. Additionally, targeting glutamate receptors, particularly the N-methyl-D-aspartate receptor, offers potential for neuropathic pain treatment. Disrupted glutamate homeostasis in MS leads to elevated levels or altered uptake of glutamate, contributing to CNS tissue damage and increased pain sensitivity. Glutamate receptor antagonists and drugs enhancing glutamate transporter activity have demonstrated efficacy in limiting motor symptoms and reducing pain in MS animal models [28].

Ongoing research into MS treatments encompasses a wide array of approaches aimed at managing the disease and improving patient outcomes. Hematopoietic stem cell transplantation has been studied since the 1990s for refractory relapsing MS to reduce autoreactive lymphocytes, showing potential benefits, especially in younger patients, though outcomes vary. Similarly, Bruton’s tyrosine kinase inhibitors such as masitinib, tolebrutinib, and evobrutinib are under study, targeting B-cell maturation to modulate immune responses in both relapsing-remitting MS and progressive MS. Research on remyelination and myelin repair continues with agents such as opicinumab, clemastine fumarate, and bazedoxifene acetate to restore CNS function, despite clinical challenges. Emerging treatments are also exploring the microbiome's impact on MS and the potential of neuromodulation techniques to manage symptoms [45,46].

The advancement of digital applications and remote communication technologies has revolutionized MS care, benefiting both patients and healthcare professionals. The Barts MS Blog, established in 2009, serves as a model for disseminating evidence-based information about MS [47]. Web-based programs such as My MS Toolkit and personalized patient support programs enhance symptom management and medication adherence [48]. Mobile apps offer various functions, including symptom tracking, education, social networking, and rehabilitation support for MS patients. Motion wearable devices show promise for assessing walking behavior and other aspects of daily life in people with MS, although limitations exist [49].

## Conclusions

MS is a complex autoimmune disorder characterized by its effects on the CNS, leading to a wide array of symptoms, including significant neuropathic pain. The pathophysiology of neuropathic pain in MS is primarily attributed to myelin damage, inflammation, and the resultant disruption of central sensory pathways. This type of pain presents uniquely challenging management issues due to its diverse manifestations and the mixed efficacy of conventional pain treatments. The integration of clinical evaluations, advanced imaging, and comprehensive symptom assessments remains critical for accurately diagnosing and effectively managing neuropathic pain in MS patients. Treatment strategies continue to evolve, combining pharmacological interventions, such as anticonvulsants and antidepressants, with emerging therapies targeting underlying inflammatory processes and novel approaches such as neuromodulation and cannabinoids.

Furthermore, addressing the psychosocial aspects of neuropathic pain is essential, as it profoundly impacts patients' quality of life. This holistic approach underscores the importance of multidisciplinary strategies in managing MS, emphasizing personalized patient care to enhance outcomes and improve overall well-being. As research progresses, the future holds promise for more targeted and effective therapies that address both the symptoms and root causes of MS, with an ongoing commitment to improving the lives of those affected by this challenging disease.
